# A Survey on the Integration of Blockchains and Databases

**DOI:** 10.1007/s41019-023-00212-z

**Published:** 2023-04-24

**Authors:** Changhao Zhu, Junzhe Li, Ziyue Zhong, Cong Yue, Meihui Zhang

**Affiliations:** 1 Beijing Institute of Technology, Beijing, China; 2 National University of Singapore, Singapore, Singapore

**Keywords:** Blockchains, Databases, Data management, Introductory and survey

## Abstract

The success of blockchain technology in cryptocurrencies reveals its potential in the data management field. Recently, there is a trend in the database community to integrate blockchains and traditional databases to obtain security, efficiency, and privacy from the two distinctive but related systems. In this survey, we discuss the use of blockchain technology in the data management field and focus on the fusion system of blockchains and databases. We first classify existing blockchain-related data management technologies by their locations on the blockchain-database spectrum. Based on the taxonomy, we discuss three types of fusion systems and analyze their design spaces and trade-offs. Then, by further investigating the typical systems and techniques of each type of fusion system and comparing the solutions, we provide insights of each fusion model. Finally, we outline the unsolved challenges and promising directions in this field and believe that fusion systems will take a more important role in data management tasks. We hope this survey can help both academia and industry to better understand the advantages and limitations of blockchain-related data management systems and develop fusion systems that meet various requirements in practice.

## Introduction

Blockchain technology has come into people’s view with the release of the Bitcoin white paper [1] in 2008. Since then, more and more cryptocurrencies and decentralized applications are adopting blockchain technology. Blockchain has taken the world by storm in the past decades.

The huge success of blockchain technology raises people’s interest in applying it to the data management field. A blockchain is essentially a novel data management system, which is maintained by multiple participants (or nodes). Compared to traditional database systems, there may be some participants behaving unexpectedly, but blockchains hold some promising properties under such a circumstance to protect the integrity of data.

- *Decentralization.* There is no central node in a blockchain system and every node in the network holds a replica of the data. In this way, the blockchain eliminates the risks that come with a centralized storage schema in traditional databases, i.e., malicious or failed central storage may cause the loss of data.
- *Immutability.* Once data are appended to the blockchain and confirmed by the majority of the chain’s participants, it can never be replaced or reversed as the records are linked one after another with hash values. This marks blockchains as different from regular databases, in which information can be easily edited or deleted.
- *Tamper-Proof.* When mining a new block, metadata of current system states and corresponding proofs are generated and distributed to the network with the replication of the block. Since the proof is guaranteed by cryptography methods, any tiny alteration to the data will lead to a failure of validation. If there is any conflict during block validation, the participant can immediately recognize that the block has been tampered with, then he can refuse this block to protect the security of the data.
- *Provenance.* Since the immutability of blockchain, the only accepted way to modify what has already been on the chain is to create a new log and append it to the chain to declare the invalidity of previous data. This mechanism ensures that every modification of data entry can be recorded as a trail, from which one can clearly obtain the history status of the data.

Despite the strong guarantee in data security, blockchain is still far from an ideal data management system. It suffers from low performance, high resource consumption, and potential privacy concerns.

- *Performance.* With its underlying chain structure, blockchain has to process each transaction serially. Moreover, other participants validate the received block by replaying the transactions in it, which is also a sequential process. These two linear transaction processing steps have a significant impact on the blockchain’s performance. It is reported that Bitcoin, as a representative blockchain system, only achieves a throughput of 7 transactions/second. In contrast, a commercial database system can easily process 2000 to 56,000 transactions in one second [2].
- *Resource Consumption.* On one hand, as the transactions go on, the append-only ledger consumes more and more storage, which will be a burden for devices with limited storage capacity such as smartphones or even personal computers. On the other hand, the mining procedure requires participants to compete with others to calculate a specific problem, while only one of them wins the right to append a block, which wastes massive energy and computing resources.
- *Privacy Issues.* Every participant in a blockchain network holds the full copy of data due to the verification need. However, this is at the cost of some privacy concerns. In real-world business applications, companies will never want collaborators or customers to access their sensitive information, while this goal can be easily achieved by leveraging views in databases.

Apparently, blockchain technology has its superiority and defect, and neither it nor a database can perfectly undertake all the requirements of modern data management tasks. Fortunately, blockchains and databases share so many similar technical concepts and solutions, making it possible to combine the strengths of security, efficiency, and privacy from both sides. For example, transactions in both systems result in state changes and should hold ACID properties to ensure their reliability. Smart contracts in blockchains are corresponding to stored procedures in databases, as they aggregate transactions. Moreover, both systems adopt indexes to satisfy various requirements, i.e., tamper-proof and verifiability for blockchains, and efficient query for databases.

We have noticed that there are massive works trying to integrate blockchain and database technologies to develop a fusion system that protects data integrity and processes transactions effectively at the same time. Though the integration of blockchains and databases has attracted more and more attention, there are few discussions about it. At present, most of the surveys about blockchains [3–9] concentrate on some specific domains, instead of a comprehensive study of the trend of fusion. We argue that drawing a whole picture is of vital importance, as it will better guide the database community to develop systems that fit various real-world needs.

*Difference with Existing Works* Existing surveys only focused on some specific aspects of blockchains in the data management field. For example, Wang et al. [6] investigate the storage and query techniques of blockchains, while the authors of [5, 8] focus on the applications in specific domains. Other aspects including system architecture [3], query processing [4], and sharding technique [7, 9, 10] have also been analyzed. There are also surveys [11, 12] trying to comprehensively analyze blockchains as a data management system; however, their goal is to dichotomize blockchains and databases, and to compare the two systems.

The trend of the fusion between blockchains and databases has also been noticed and analyzed in other works. Based on the comparisons between blockchains and distributed databases, Ruan et al. [13] discuss the fusion trend and some representative works. Recently, the authors of [14] conduct extensive experiments on some hybrid blockchain database systems and reveal the variety of design choices of such systems. However, due to the experimental limitations, only a few systems are studied. Thus, some most recent works are not covered and discussed.

*Contributions* In this paper, we conduct a comprehensive survey on the integration of blockchains and databases in the data management field. To sum up, we made the following contributions.

- We propose the blockchain-database spectrum, a framework to analyze the works about blockchains in the data management field, and recognize the trend of integrating blockchains and traditional databases. We further identify three typical models of the fusion, namely *database-oriented blockchains*, *blockchain-oriented databases*, and *hybrid systems*, and conduct a comprehensive comparison of the three types of systems in the design spaces and trade-offs.
- We review each of the representative systems of *database-oriented blockchains*, *blockchain-oriented databases*, and *hybrid systems*. Besides, we summarize and evaluate the techniques used in each model, which provides insights into each fusion model.
- Based on the exhaustive research and analysis of existing works, we discuss the limitations of existing methods for blockchain-related data management systems and provide future research directions.

The rest of this paper is organized as follows. First, the preliminaries are provided in Sect. 2, including a basic introduction to blockchains and databases and the blockchain-database spectrum. We also classify existing blockchain-related data management technologies by their coordinates on the blockchain-database spectrum and identify their design spaces in this section. We review the representative systems and techniques of *database-oriented blockchains*, *blockchain-oriented databases*, and *hybrid systems* in Sects. 3–5, respectively. Then, we compare these systems and provide challenges and opportunities in the blockchain-related data management field in Sect. 6. Finally, Sect. 7 concludes the paper.

## Preliminaries

### Backgrounds

We begin this section with some basic information about blockchains and databases to provide a primary impression of the two different but relevant technologies.

**Fig. 1:**
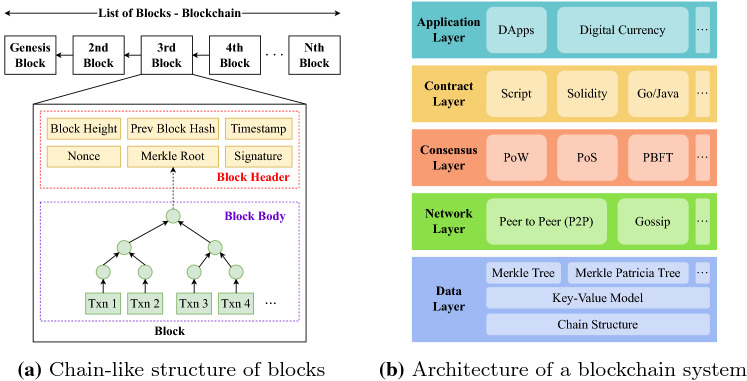
Blockchain overview

#### Blockchain

Blockchain is an innovative data storage and management technology that integrates a variety of established technologies, including high-performance data storage, peer-to-peer networks, cryptography, consensus protocols, etc. The concept of blockchain originated from Bitcoin, which is proposed by Satoshi Nakamoto [1], and most of the existing blockchain systems also follow the chain structure in Bitcoin. Taking Bitcoin as an example, the structure of a typical blockchain is shown in Fig. 1a. Blocks are connected in a linked list and new blocks can only be added at the end of the chain. Therefore, all nodes in the blockchain system store the blocks and the transactions in a consistent order. The block, the basic structural unit of the blockchain, consists of a block header containing metadata and a block body containing transaction data. Block header consists of block height, the previous block’s hash value, timestamp, nonce, miner signature, and Merkle root, and block body can be viewed as a collection of transaction records consisting of multiple transactions. For example, the block body of the Bitcoin system contains a Merkle tree consisting of approximately 2,500 transaction records that have been hashed, each consisting of information such as transaction hash, inputs, outputs, timestamps, and fees. Blocks are connected by hash values. The hash value of each block is obtained by re-hashing the Merkle tree’s root, the previous block’s hash value, and other information. Any change in transaction data in a block will cause a change in the hash value of this block, which in turn will change all subsequent blocks along the chain. To sum up, blockchain incorporates the hash function in the chain structure, making data tampering infeasible in blockchain and enhancing data storage security.

For a clear understanding of the blockchain hierarchy, we abstract the blockchain into 5 layers in Fig. 1b.

- *Data layer*. To efficiently organize various data in the blockchain, the data layer contains elements such as data structure, transaction model, index data, state data, and persistent storage scheme.
- *Network layer*. To meet the communication between nodes in a decentralized blockchain network, the P2P protocol plays an important role in the network layer. The content transmitted between nodes mainly consists of transaction data and block data.
- *Consensus layer*. Unlike centrally governed databases, blockchain uses a distributed consensus algorithm to ensure that nodes in the network that do not trust each other can agree on the same ledger. The use of consensus algorithms improves the blockchain’s ability to cope with crash tolerance or Byzantine fault tolerance, giving the blockchain a higher level of security than traditional databases.
- *Contract layer*. Containing various scripts, algorithms and smart contracts, it is the foundation of blockchain programmability.
- *Application layer*. Users can easily develop new decentralized and cryptographically secure blockchain-based applications using the APIs provided by the blockchain.

*Permissionless and Permissioned* Blockchains can be broadly classified into two categories: permissionless blockchains and permissioned blockchains.

Permissionless blockchains are a type of blockchain in which anyone can participate in the network without any prior approval or authorization. It is often referred to as a public blockchain as the network is open to the public. Examples of popular permissionless blockchains include Bitcoin, Ethereum [15], etc. Ethereum abandons the UTXO transaction model proposed by Bitcoin in favor of the account/balance transaction model and extends the Merkle tree to the Merkle Patricia Trie (MPT) [16]. The Merkle roots originally stored in the block header are changed to three Merkle Patricia tree roots in Ethereum, corresponding to the world state tree, transaction tree, and receipt tree, respectively. Besides, the most important innovation of Ethereum is that it provides Turing-complete scripting languages Solidity and Serpent, and provides a sandbox environment Ethereum Virtual Machine (EVM) for users to write and run smart contracts. With programmability, built-in persistent state storage, and Turing completeness, smart contracts make it easy for developers to create their blockchain applications on the Ethereum platform, which marks the birth of the blockchain 2.0 era.

Permissioned blockchain are blockchains that require permission to join and participate in consensus. Hyperledger Fabric [17], a permissioned blockchain, has evolved into an enterprise-accessible distributed ledger technology platform. Fabric allows users to write smart contracts (also called chaincodes) in Go or Java. Therefore, it is the first platform that supports high-level programming languages for writing smart contracts. Unlike permissionless blockchains such as Bitcoin and Ethereum, Fabric adds an access mechanism where only authorized nodes can join the network and uses Raft, an efficient algorithm but cannot resist Byzantine behaviors. Fabric is innovative in that it uses a loosely coupled design that modularizes consensus algorithms, authentication, key management protocols, and cryptographic libraries, further meeting the diversity of enterprise needs for blockchain.

*Innovations on Blockchain* Blockchains have achieved great success and promoted many developments in different fields. However, traditional blockchain systems still suffer from problems of low throughput and high latency. There are several innovations in consensus algorithms and transaction concurrency to address these issues.

The consensus algorithm is one of the core technologies of blockchain, which describes how the peers reach an agreement on the state of the world. The efficiency of consensus algorithm impacts the performance of the entire blockchain system. Here, we introduce some BFT-based protocols. Castro et al. [18] propose PBFT consensus algorithm which reduces BFT’s complexity from exponential to polynomial. To further optimize the decentralization level and performance scalability of blockchains, SBFT [19] reduces communication to linear with collectors and threshold signatures. FastBFT [20] designed a novel message aggregation technique, reducing message complexity from $O(n^2)$ to *O*(*n*).

The purpose of concurrency control is to optimize transaction processing, which involves improving the efficiency of transaction validation, execution, and confirmation on blockchains. Take Hyperledger Fabric for instance, although it parallelly executes the transactions in the execution phase, the throughput cannot further improve, especially when there are high contentions among each transaction. To be more specific, though all the conflict transactions are simulated in the execution phase, only one of them can be eventually submitted in the final validation phase, and others have to be aborted. The solution is to reduce the abort rate. Fabric++ [21] uses reordering and early abortion to solve this problem. It obtains the read/write set of each transaction in the execute phase and recognizes the conflicting transactions with a dependency graph between the transactions within the same block. Then it reorders the transactions and early aborts the transactions that cannot be serialized. FabricSharp [22] optimizes the reordering mechanism to support inter-block transactions, which further promotes the commit rate and the performance in terms of throughput. FastFabric [23] extends the concurrency of Fabric by introducing a validation pipeline, which parallelizes as many validation steps as possible, including endorsement policy validation and syntactic verification. Finally, XOX Fabric [24] proposes a novel hybrid execution model consisting of a pre-order and a post-order execution step which makes a trade-off between minimal invalid transactions and maximal concurrent execution.

#### Database

Database technology has been developed for decades. Unlike blockchain, it supports features like ACID properties, complex queries, low transaction latency, high throughput, and scalability. Mainstream databases are divided into three categories: SQL databases, NoSQL databases, and NewSQL databases.

- *SQL databases*. As one of the most widely used databases supporting the relational model, SQL database (e.g., MySQL [25], Oracle [26]) is usually used to store structured data which is highly-organized and formatted. Therefore, SQL databases can comply with atomicity, consistency, isolation and persistence. In addition, SQL databases also have good support for transaction concurrency control and data privacy protection.
- *NoSQL databases*. To have better horizontal scalability, many databases abandon the relational model and support for SQL statements, replacing them with support for semi-structured and unstructured data. These databases are called NoSQL databases. Unlike relational databases, NoSQL databases have multiple types: key-value databases (e.g., LevelDB [27], BerkeleyDB [28], Redis [29]), column-oriented databases (e.g., Bigtable [30], Apache HBase [31]), document-oriented databases (e.g., MongoDB [32]), graph databases (e.g., Neo4j [33]), time series databases (e.g., InfluxDB [34]) and so on.
- *NewSQL databases*. A new type of database management system (DBMS) is designed to provide a NoSQL system’s high scalability and performance while retaining the ACID transactional characteristics of a traditional relational database management system (RDBMS). NewSQL systems can use both relational and non-relational data models. The mainstream NewSQL systems include Google Cloud Spanner [35], CockroachDB [36], TiDB [37], and Amazon Aurora [38]. These NewSQL systems are all built with distributed architectures that provide high scalability and performance while retaining ACID transaction features and SQL query language support. Their emergence provides new options for addressing the needs of large-scale data processing and distributed systems.

In general, blockchains and databases are different data management technologies with different futures and application scenarios. Blockchains have the advantage of security for applications requiring security, while databases have the advantage of performance and usability for large-scale data processing and high concurrent access.

### Blockchain-Database Spectrum

**Fig. 2:**
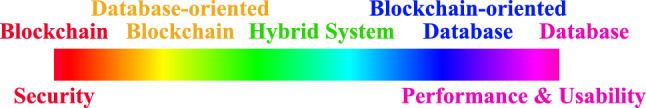
Blockchain-database spectrum

Though blockchains and databases are essentially designed for different goals, both systems have the capability to manage data. Along this point of view, we present our blockchain-database spectrum in Fig. 2 to compare them and find possible fusion directions.

In this framework, blockchains lie at the security end of the spectrum, while databases are at the other performance end. Besides both ends, there are also systems located in the middle parts of the blockchain-database spectrum. These systems are fusions of blockchains and databases to varying degrees and can be further classified into three major types, namely *database-oriented blockchains*, *blockchain-oriented databases*, and *hybrid systems*. As Fig. 2 depicts, the difference lies in the design considerations and trade-offs between performance and data security.

### Fusion Systems

In this survey, we focus on the fusions systems, i.e., *database-oriented blockchains*, *blockchain-oriented databases*, and *hybrid systems*, which occupy the middle parts of the blockchain-database spectrum in Fig. 2. We will informally define these systems and briefly describe their design considerations in this section, while leaving the details to the rest of this survey. A high-level and coarse-grained comparison between pure blockchains, pure databases, and three types of fusion systems based on their locations in the blockchain-database spectrum is summarized in Table 1.

**Table 1:** A high-level comparison of inherent features between data management technologies

System	Security			Performance and usability				
	Decentralization	Data security	Auditability	Performance	Ease of Use	Data Model	Privacy	Resource requirement
Blockchains	High	Very high	Every participant	Very low	Low	KV	No	Very high
Database-oriented Blockchains	High	Very high	Every participant	Low	Medium	KV [39, 40], Relational [41, 42], Document [43]	Low	High
Hybrid Systems	Partial	High	Every participant	Low	High	Relational [44], Document [45], Graph [46]	Low$^{\hbox {a}}$	High
Blockchain-oriented databases	Partial	High	Authorized users	High	High	Relational [47–51], Column [52]	High	Normal
Databases	No	Basic	Authorized users	Very high	High	KV, Relational, Graph, Document	High	Normal

$^{\hbox {a}}$No privacy for the on-chain metadata

*Database-Oriented Blockchains* The *database-oriented blockchains* are at the blockchain side of the blockchain-database spectrum. Same as blockchains, *database-oriented blockchains* retain the essential chain-like structure of ledgers, which keeps track of data modifications and ensures data security. Besides the security, *database-oriented blockchains* also pursue features to provide a better experience in real-world practice just as databases do, such as easy-to-use APIs, higher throughput, lower resource consumption, and assurance of secret data’s privacy. To sum up, *database-oriented blockchains* are a collection of systems that are built on top of blockchains and integrated with database features.

As it has been revealed in the spectrum, the most straightforward and widely-used solution is to equip the systems with mature techniques from databases, including sharding [7, 53–59], concurrency control [21, 49, 60–64], indexing [41, 62, 65–75], views [76, 77], and so on. There is another direction that modifies existing components in blockchains, such as consensus protocols [50, 78–81] and data processing layers [41, 82–85].

*Blockchain-Oriented Databases* Opposite to the *database-oriented blockchains*, the *blockchain-oriented databases* are closer to databases. Such systems pay more attention to processing performance and usually support more complicated data models such as relational. Some of them also support SQL-like interfaces, making them more convenient for application developers.

To achieve such a goal while keeping a basic security guarantee, *blockchain-oriented databases* are built upon an existing database instance, while learning lessons of hash chain from blockchains. That is, they usually contain a blockchain layer [50] or a middleware [48, 49] with blockchain features, and the chain-like relationships are either revealed by the internal fields or stored in a specific table. We regard such systems as general *blockchain-oriented databases* and introduce the technical details in Sect. 4.

We also notice that there is another way to build a database system that supports verifiable data processing, which results in the so-called ledger databases [86–89]. However, such systems adopt a different trust assumption with blockchains, i.e., there is usually a centralized service provider and require a trusted auditor to replay the log of transactions to detect if the server has tampered with the data. Hence, we exclude the ledger databases from the *blockchain-oriented databases*, and ignore them in the following of this paper.

*Hybrid Systems* Such systems locate around the very center of the spectrum, which means they reach a balance between security and performance. Note that this can be interpreted into two situations. The ideal one is to achieve decentralized data security as blockchains and high throughput as commercial databases at the same time. However, this is an unreachable target at present and no one has been recognized to provide a perfect solution to this problem. On the other hand, equally combining blockchains and databases into a single system is also a way to reach the balance [44–46]. This usually results in a middleware that connects a blockchain of metadata or logs, and a database of various forms of data. In this way, such systems ensure the security of metadata and the performance of data processing, which is at the cost of inheriting some defects from both sides. For example, the system may be redundant to include both instances, and the actual data stored in the database are usually not protected by the blockchain. We use *hybrid systems* to refer to the latter systems in the rest of this survey.

## Database-Oriented Blockchains

The efforts to explore the data management possibility of blockchains have taken a long way. At the early stage of the exploration, many researchers try to adopt blockchains to real application scenarios, which leads to the earliest *database-oriented blockchains*. For example, MedRec [90] is an Ethereum-based decentralized system to process electronic medical records (EMR) and can be integrated with the existing EMR management systems. It utilized the data management ability of underlying Ethereum by proposing three dedicated smart contracts to contain metadata about the record ownership, permissions, and data integrity. Other works attempt to apply blockchain to other fields and manage corresponding data with the help of smart contracts, such as vehicles [91], cognitive radio (CRs) [92], IoT [40], cloud computing and services [93–96], decentralized privacy-preserving search [97], MOOC [98], and COVID-19 contact tracing [99].

However, the aforementioned systems just take exiguous steps toward databases in the spectrum. The successors propose prototype systems or protocols to manage general data with integrated database systems, which mainly focus on data integrity. Gaetani et al. [100] design a two-layer blockchain-based system in cloud computing environment. The first layer uses a lightweight distributed consensus protocol that ensures low latency and high throughput, and the second one is a PoW blockchain to ensure data integrity. Sui et al. [101] propose an encrypted data management system with mandatory access control, in which blockchain provides the integrity guarantee. Konashevych proposes a protocol [39] to design a cross-blockchain database that manages data on different chains and solves problems of immutability, as well as duplication of tokens as the result of hard forks.

**Fig. 3:**
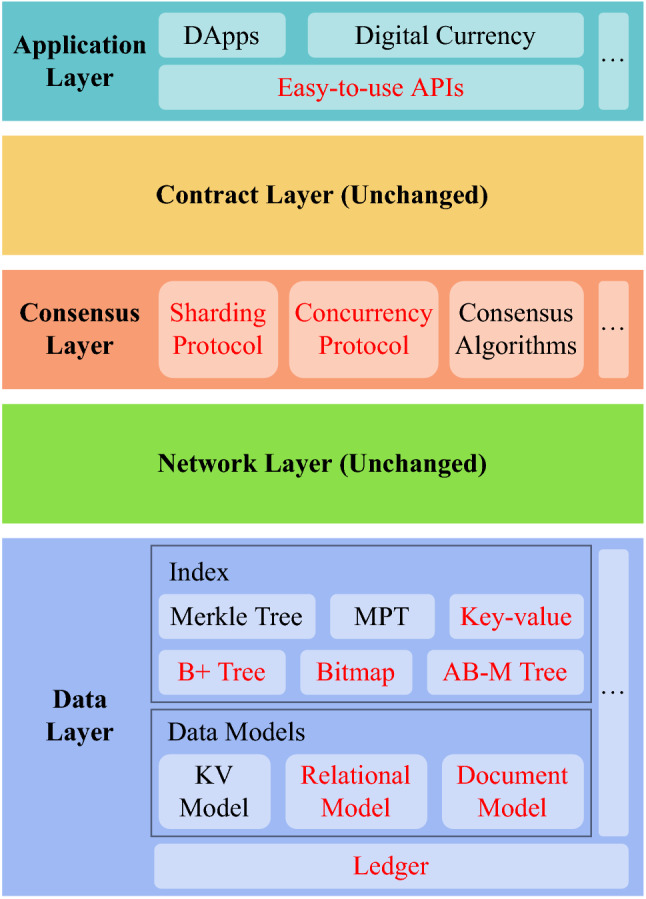
Architecture of *database-oriented blockchains*

Recently, researchers of *database-oriented blockchains* aim to equip pure blockchain systems with the ability to manage general data and reach the goal of high throughput, low resource consumption, easy-to-use APIs, and privacy of secret data. Such systems usually modify several components of blockchains, including: (1) index, (2) protocol, e.g., sharding and consensus, (3) API and data models, and (4) ledger arrangement, as Fig. 3 shows. Note that these technical routes do not necessarily correspond to the goals. They can either be combined to solve a single problem, or improve the system in various aspects individually. Moreover, many researchers try to improve multiple aspects in their single system and adopt many techniques. Thus, we review existing studies from the technical routes rather than the goals in this section.

### Index

In databases, an index is a structure that sorts the specified values which aim to boost query processing and data updates. However, indexes in blockchains usually take an additional task to prove the integrity of data as an authenticated data structure (ADS) does. For example, Ethereum uses MPT to index the states of each account and protect the data. However, such an index has poor performance since it has to fetch data from LevelDB whenever it visits a node in the MPT. Thus, recent works try to develop indexes that fit the batch data in the blockchain environment, which improves the performance of the indexes of *database-oriented blockchains*. Besides the original data, researchers also index some metadata to support a broader range of queries. There are also works trying to add concurrency to the indexes that support parallel updates.

The efforts around the indexes are summarized in Table 2 and introduced in detail as follows.

**Table 2:** Summary of representative indexes in *database-oriented blockchains*. For the general notations, *n* refers to the number of blocks, *N* refers to the number of transactions, and *e* refers to the number of data entries

System	Index	Underpinning techniques	Index Level	Query time complexity	Space occupation	Supported query types$^{\hbox {a}}$
SEBDB [41]	B$^+$ Tree Index	B$^+$ Tree	Block	$O(\log {n})$	*O*(*n*)	SQL-like (condition), Provenance, On-chain Join, On-off Join
	Bitmap Index	Bitmap	Table	*O*(1)	*O*(*n*)	
	Layered Index	Bitmap, B$^+$ Tree	Transaction	$O(\log {N})$	*O*(*bN*)$^{\hbox {b}}$	
AuthQX [69, 70]	–	Merkle B Tree, SkipList	Entry	$O(\log {e})$	*O*(*e*)	Range
SE-Chain [72]	AB-M tree	Balanced BST, Merkle Tree	Transaction	*O*(*N*)	*O*(*N*)	KV
Yan et al. [67]	B$^+$ Tree Based Index	B$^+$ Tree, Bitmap	Block	$O(\log {n})$	*O*(*n*)	KV, Range
	Key-valueIndex	Key-value	Transaction	*O*(1)	*O*(*N*)	KV
ForkBase [73]	POS-tree	B$^+$ Tree, Merkle Tree	FNode	$O(\log {m})$ $^{\hbox {c}}$	*O*(*m*)	KV
LineageChain [74, 75]	DASL	SkipList	State (Transaction)	$O(\log {N})$	*O*(*N*)	KV, Provenance
vChain [65]	Intra-block Index	Merkle Tree	Transaction	$O(\log {N})$	*O*(*N*)	Boolean, Range$^{\hbox {f}}$, Subscription
	Inter-block Index	SkipList	Block	$O(\log {n})$	*O*(*n*)	
	IP-Tree	Prefix Tree, Inverted File	Grid Node	*O*(*l*)$^{\hbox {d}}$	*O*(*k*)$^{\hbox {e}}$	
vChain+ [68]	SWA Index	Sliding Window, Merkle Tree	Entry	*O*(*w*) $^{\hbox {g}}$	*O*(*w*)	Range, Multi-dimensional, Combination
Zhu et al. [85]	GCA$^2$-tree	Merkle Tree	Block	$O(\log {n})$	*O*(*n*)	Multi-dimensional Aggregation (Count, Max, Min, Average)
Feng et al. [62]	Merkle Forest	Merkle Tree	Entry	$O(t\log {e})$ $^{\hbox {h}}$	*O*(*e*)	Merkle Multiproofs
Zhang et al. [102]	GEM$^2$-Tree	SMB Tree, Merkle B Tree	Entry	$O(\log {e})$	*O*(*e*)	Range
Zhang et al. [103]	Suppressed Merke$^{inv}$ Index	Merkle B Tree	Entry	$O(\log {e})$	*O*(*e*)	Keyword
	Chameleon$^{inv}$ Index	Chameleon Tree	Entry	$O(\log {e})$	*O*(*e*)	
	Chameleon$^{inv*}$ Index	Chameleon Tree, Bloom filter	Entry	$O(\log {e})$	*O*(*e*)	

$^{\hbox {a}}$We omitted the “authenticated” or “verifiable” prefixes, since they are already guaranteed by the blockchains

$^{\hbox {b}}$ *b* is the number of buckets that describes the distribution of attribute’s values among blocks

$^{\hbox {c}}$ *m* represents the number of FNodes, which is specific to the queries

$^{\hbox {d}}$ *l* represents the number of grid node layers, which is specific to the queries

$^{\hbox {e}}$ *k* represents the number of all the grid nodes, which is specific to the queries

$^{\hbox {f}}$ Only supports integers, fixed-point numbers, or other data types that can be transformed to set-valued attributes

$^{\hbox {g}}$ *w* is related to the user-defined sliding window size, thus the overhead is constant to a given sliding window size

$^{\hbox {h}}$ *t* is a user-defined parameter to control the layer of the Merkle Forest

#### Boosting Data Access

The authors of SEBDB [41] identified three basic operations in blockchains, namely: (1) fetching a block by a given block id, transaction id, or timestamp, (2) fetching tuples with the same transaction type, and (3) fetching transactions by given conditions. They designed a corresponding index structure for each operation to boost data access. For the first operation, a block-level B$^+$ tree with key (block_id, first_tx_id, ts) is built. In this way, given the query condition, one can go from the root down to the leaf node to get the location of the target block. A table-level bitmap index recording table distribution is built to solve the second scenario. Each bitmap refers to a table, and the i-th bit in a bitmap indicates whether block i contains transactions of that table or not. Layered indexes are designed to deal with the third operation, in which the first level consists of bitmaps or entries that describe the distribution of attribute’s values among blocks, while the second level is a B$^+$ tree for the attributes within the block.

AuthQX [69, 70] runs in an environment with TEEs, which provides an isolated memory for sensitive data and ensures secure computing in the hardware level. However, the limited memory of TEE hinders its wide application. To solve this problem, the authors developed a mechanism to organize data hierarchically in the untrusted and trusted memory and designed corresponding index structures. To be more specific, data in the untrusted memory are organized into Merkle B trees, while the frequently accessed internal nodes are cached in the TEE. The skip list maintained in trusted memory buffers newly attached block data. Once the capacity of the skip list reaches the threshold, the merge operation from the skip list to the MB tree will be started. The replacement between hot and cold data follows LRU strategy. What’s more, since the data cached in the TEE are authenticated, the update inside the TEE can be batched to improve the efficiency when the tree is frequently updated.

Each transaction of SE-Chain [72] is maintained in the AB-M tree (adaptive balanced Merkle tree), which combines the advantages of balanced binary trees (fast retrieval) and Merkle trees (fast verification). Specifically, an AB-M tree is divided into two layers, the lower one is a Merkle tree, and the upper one is a binary tree containing node hash information, so as to meet the verification requirements from leaf node to root node. There is a threshold *T* controlling the size of the Merkle tree, while the rest are arranged in the binary tree. When processing queries, the system first searches an approximate range from the top level according to the balanced binary tree search algorithm, and then traverses the Merkle tree to fetch the specified data.

Yan et al. [67] designed a dual-index to adapt the block form of data storage for their proposed construction engineering management system. Instead of traversing the entire blockchain, there is a B$^+$ tree-based index and a key-value index in the proposed system to accelerate the queries. The former one is arranged by the locations, and takes care of range queries. The internal nodes of the B$^+$ tree index contain a bitmap to indicate whether there is a file of specific types, which boosts the queries by the file types. The keys of the latter index are hashes of each file and the values are the corresponding transactions, which is responsible for the single queries.

#### Enriching Query Types

With the help of specifically designed indexes for basic blockchain operations, SEBDB [41] further supports SQL-like operations such as track-trace, on-chain join, and on-chain and off-chain join (on-off join). The roles of the proposed indexes are described as follows. First, a track-trace operation is to find who sends the transaction and which transaction is done. To support this, the layered indices on the operation sender and operation type are pre-created. Second, for the on-chain join, the table-level index can accelerate the searching process, and layered indexes on the join attribute can further optimize the performance. Third, similar to the on-chain join situation, the bitmap index and layered index on the join attribute also help the scan procedure of on-chain data.

The structures of most blockchains’ index not only depend on the items stored in the index, but also on its update history. However, the authors of ForkBase [73] extracted the need for structurally-invariant reusable indexes (SIRI), whose structure is uniquely determined by the set of records. They further proposed a SIRI instance called POS-Tree. In a POS Tree, the data entries are sorted and arranged into a byte sequence. Then, different types of split functions are applied to the sequence recursively to create leaf nodes and internal nodes, which are modeled as FNodes in the POS-Tree. The FNodes are linked and protected as those in Merkle trees. In this way, the POS-Tree supports effective data deduplication of multi-version data, which enables fork semantics of blockchains that manage the conflicts.

LineageChain [74, 75] supports online forward provenance tracking, i.e., providing historical blockchain states in a tamper-evident manner to smart contracts while they are running. To achieve such a goal, LineageChain reorganizes the leaf nodes in the original Merkle tree into a Merkle DAG, to enhance the storage layer of blockchains, and provide efficient tracking and tamper evidence. Then, it indexes the Merkle DAG with a deterministic append-only skip list (DASL) to avoid searching from the head of DAG. The DASL leverages the append-only and non-random properties of blockchains to distinguish it from normal skip lists. Such a scheme enables fast and low-cost history data query, making it possible to track a specific value when a smart contract is running.

There are light nodes that only store block headers in a blockchain network, and they usually represent a user. It is important for them to verify the integrity of query results. Xu’s team successively proposed systems to support authenticated queries for light nodes [65, 68]. These systems split the indexing and proving function of blockchain indexes and designed accumulator-based ADSs. In vChain [65], an accumulator-based ADS is proposed to aggregate any query attributes dynamically and support authenticated Boolean queries, while a Merkle tree-based intra-block index and a skip list-based inter-block index undertake the acceleration task. The authors also build an inverted prefix tree (IP-Tree) over subscription queries to efficiently handle a large number of subscription queries. By introducing a set accumulator-based ADS with sliding time window and building corresponding SWA index, vChain+ [68] supports authenticated queries on range, multi-dimensional, and the combination of these types. Zhu et al. proposed another accumulator-based ADS, namely GCA$^2$-tree [85], that supports verifiable multidimensional aggregate queries. To enrich authenticated query types in the hybrid-storage blockchain, Zhang et al. [102] propose a gas-efficient ADS, called GEM$^2$-tree, which supports authenticated queries. To further reduce gas cost due to storing intermediate data in GEM$^2$-tree and extend keyword search in hybrid-storage blockchain, Zhang et al. [103] design novel ADS schemes, such as suppressed Merkle$^{inv}$ index, Chameleon$^{inv}$ index, and Chameleon$^{inv*}$ index.

#### Adding Concurrency Support

Fang et al. [62] focus on introducing concurrency to blockchains. Besides the concurrency in the transaction execution framework, they also upgrade the index to support parallel updates and validations. Specifically, they designed a Merkle Forest consisting of multiple sub-trees at a specified size of $2^N$ to increase the parallelism of generating multiproofs for data and verifying data integrity. It is a layered structure that the roots of the lowest level are the leaves of the upper level, and the root is computed recursively. In this way, the modification of low-level Merkle trees can be done in parallel, and the recursive updates of upper-level trees are batched to decrease the overhead. The validation of the multiproofs is conducted from bottom to top.

### Protocol

In blockchains, protocols are a set of rules that allow participants to communicate and share data. Though the existing blockchain protocols ensure relatively secure communication, the full-replicated and serial nature lowers the whole system’s performance, which hinders the further application of blockchains in the data management field. In this survey, we focus on two of the promising solutions, namely sharding and concurrency. In addition, the consensus algorithm is orthogonal with the two approaches and can be arbitrarily combined with them according to actual needs. We provide an overview of the surveyed works in Table 3.

**Table 3:** Summary of representative works about protocols in *database-oriented blockchains*, in which “A/B” refers to the Account/Balance model

System	Type	Threat model	Transaction model	Consensus protocol	Fault tolerance	Throughput$^{\hbox {a}}$	Additional techniques
Elastico [104]	UTXO	Permissionless	BFT	PBFT	1/3 (Intra-shard) 1/4 (Total)	N/A	–
OmniLedger [105]	UTXO	Permissionless	BFT	ByzCoinX	1/3 (Intra-shard) 1/4 (Total)	13,000	2PC
RapidChain [106]	UTXO	Permissionless	BFT	PBFT+EC	1/2 (Intra-shard) 1/3 (Total)	7380	Erasure Coding
Monoxide [107]	A/B	Permissionless	BFT	Chu-ko-nu Mining	1/2	11,694.89	–
SlimChain [61]	A/B	Permissioned	CFT	Raft	N/A	1284	TEE, Serializable Snapshot Isolation, Optimistic Concurrency Control
		Permissionless	BFT	PoW	1/2	462	
BrokerChain [56]	A/B	Permissionless	BFT	PBFT	1/3 (Intra-shard) 1/4 (Total)	30,000	–
Meepo [58]	A/B	Permissioned	BFT, BFT/CFT$^{\hbox {b}}$	*Any*	–	124,583.7	–
BFT-Store [53–55]	A/B	Permissionless	BFT	BFT+EC	1/r $^{\hbox {c}}$	2100	Erasure coding
Section-Blockchain [57]	A/B	Permissionless	BFT	Proof of Storage	1/2	N/A	–
SChain [63]	A/B	Permissioned	BFT	PBFT	1/3	N/A	Deterministic Concurrency Control
PEPP [60]	A/B	Permissioned	BFT	PBFT	1/3	14,000	–
SEFrame [64]	A/B	Permissioned	BFT	PBFT	1/3	N/A	Optimistic Concurrency Control

$^{\hbox {a}}$In tps (transactions per second), and each is the best throughput reported in the paper

$^{\hbox {b}}$ The inter-shard threat model is BFT, and the intra-shard one can be configured as either BFT or CFT according to the requirement

$^{\hbox {c}}$ r is configurable and specific to the BFT protocol it adopts

#### Sharding

Sharding is originally a technique in databases to expand storage capacity and reach higher throughput. By sharding, the huge data is divided into multiple subsets and stored on different nodes, so that transactions on different nodes can be processed in parallel. Many *database-oriented blockchains* also benefit from such a method and improve the data processing capability. Elastico [104] is the first sharded blockchain that divides the network into multiple groups. Each group processes disjoint transaction data and runs PBFT consensus independently. Afterward, OmniLedger [105] introduced 2PC to the cross-shard transaction process to ensure the atomicity of cross-shard transactions; RapidChain [106], combined the PBFT protocol with erasure coding (EC) [108] to reduce the huge network traffic brought by PBFT; Monoxide [107] proposed Chu-ko-nu Mining to solve the problem that the computing power in the PoW is diluted after sharding, and enhance the security of the system. However, there are still problems to be solved, e.g., the efficiency of the cross-shard transaction process, and the storage issues.

Although the transactions within the same shards can be efficiently executed in sharded blockchains, the cross-shard transactions usually become the bottleneck. Authors of BrokerChain [56] pointed out that the cross-shard transactions can be reduced if the partition can be altered. Thus, they analyze the accounts that involve in the upcoming transactions and dynamically adjust to the optimized account distribution. However, such a scheme cannot completely avoid cross-shard transactions. To solve this problem, the accounts involved in the cross-shard transactions are virtually divided into several sub-accounts with part of the assets it holds, and distributed in different shards. Thus, cross-shard transactions can be divided into intra-shard and cross-shard sub-transactions. The former can be effectively handled, while the latter is taken care of by special broker accounts in both shards.

Meanwhile, Meepo [58] provides another solution to improve the efficiency of cross-shard transaction execution. It requires a consortium environment, i.e., every node belongs to a specific organization and each organization contains several data shards. Moreover, nodes within the same organization trust each other. Under such a scenario, the authors proposed a cross-shard protocol. To be more specific, several cross-epochs are inserted after processing intra-shard transactions to execute inter-shard transactions in the consensus. Each shard sends cross-calls that include necessary data to the remote shard and executes the inter-shard transactions on the target shard. This procedure is executed repeatedly until there are no more cross-calls generated, i.e., all cross-shard transactions have been processed. This indicates the cross-epochs of the current block have finished and the consensus of the next block goes on. In this way, cross-shard communication can be done according to the order of cross-calls, which reduces the contention of cross-shard transactions and improves efficiency. Meepo also provides atomic guarantees for cross-shard transactions in replay-epoch, which follows cross-epochs to remove any faulty transactions.

Since the data are supposed to be fully replicated in the primitive blockchain network, sharding should reduce the storage overhead of every single machine. BFT-Store [53–55] is a Byzantine fault-tolerant partition storage engine that equips Reed-Solomon (RS) [109], a widely-used EC. Specifically, assuming that there are *n* nodes in the network and the system can tolerate at most *f* faulty nodes, an RS engine is responsible to encode $n - 2f$ original blocks into n chunks with $(n-2f, 2f)$-RS encoding. The choice of parameters is based on the fact that the BFT protocol can only ensure that $n - 2f$ honest nodes commit blocks. Then each chunk is distributed to a node. In this way, the storage complexity of each block is reduced from *O*(*n*) to *O*(1). The read engine handles the read requests and responses with the target block. When the target block is local to the node, it is returned directly, otherwise the node sends a query request to the target node. If the request is not replied until timeout exceeds, the node broadcasts a decoding request to $n - f$ random nodes to obtain necessary chunks and returns the decoded target block. Other components include a recovery engine that recovers the data and a scale-out engine that coordinates the re-encoding process when a new node joins the system.

Section-Blockchain [57] also tries to reduce the storage overhead without compromising the security of the system via the sharding technique. It simply partitions the blocks into several blockchain fragments and the corresponding database snapshots that record the global system settings and account states at an exact moment. Then, the author designed an efficient protocol that helps participants to optimize the connection with each other to achieve a formatted network. With such a protocol, the author proved that the data are safe when the participants hold all the block headers of the mainchain, a subset of blockchain fragments and database snapshots, and a map table between fragments and snapshots. Since the map is much smaller than a whole block, Section-Blockchain reduces the storage overhead of each participant.

SlimChain [61] adopts a novel stateless scheme, in which the off-chain storage nodes store the ledger states and simulate smart contract execution, while the on-chain consensus nodes only maintain the short commitment of ledger states. Shardings in SlimChain aim to lower the overhead of the off-chain storage nodes. In particular, each off-chain node can choose to store partial or full states based on their storage capacities. The transactions are assigned to the nodes which hold the necessary data fragment. There’s no need to worry about the cross-shard transactions that no node holds complete data, since the data can be authentically retrieved from other nodes with the TEE environment.

#### Concurrency

Many works have revealed that the serial execution of transactions is one of the bottlenecks that encumber the performance of blockchains, as it does not fully make use of the concurrency ability of modern multiprocessors. How to enable blockchains with concurrency to improve transaction execution efficiency is a hot topic in recent years, and the key lies in how to ensure that the results of concurrent schedules are the same in all nodes. As a typical blockchain system, Hyperledger Fabric [17] adopts a novel execute-order-validate (EOV) scheme, in which transactions are executed parallelly in the first stage while keeping serially in the latter two. Such a scheme inspires the design of succussing *database-oriented blockchains* to further improve the concurrency of the transactions. For example, as mentioned before, the stateless design of SlimChain [61] naturally supports the parallel execution of transactions in the off-chain storage nodes. To ensure the ACID property of these transactions, the authors introduce the concurrency control algorithms in the commit phase.

SChain [63] introduces concurrency to permissioned blockchain transactions from both intra- and inter-block. Since nodes from the same organization trust each other, transactions with the same block are assigned to different nodes and the results are shared in the organization. To maximize the concurrency while ensuring execution correctness, SChain pre-analyzes the potential conflicts by each transaction’s read/write set and assigns conflicting transactions to the same executor. As for intra-block concurrency, SChain divides the block formation into five stages and overlaps the execution process of different blocks. In other words, it turns the original pipeline model into a transaction streaming pipeline model to make the most use of the computing resources.

A parallel execution engine, PEPP [60], is proposed for the consortium blockchain. It adopts a deterministic concurrency mechanism to obtain the predetermined serial order of parallel execution, and conducts parallel update operations on the state tree. The workflow to process a transaction includes three phases, namely ordering, execution, and finalization, and the PEEP involves the latter two. In the execution phase, a schedule layer is responsible to coordinates the parallel execution in a deterministic serial order. An ordered locking mechanism is used to eliminate the non-determinism without introducing additional network communication. In the finalization phase, the results of transactions are updated to a specially designed state tree that allows deferred commits and parallel updates. In this way, the workflow will not be blocked by the time-consuming tree update, and the performance of the entire system can be further improved.

Recently, new hardware are introduced to blockchain systems. It is also important to design suitable concurrency mechanisms for these systems. SEFrame [62, 64] proposes a concurrent execution mechanism based on SGX, an instance of TEE. As mentioned before, the SGX cannot hold the entire ledger in its memory due to hardware limitations, and the data swap between it and main memory should also be minimized because of the huge cost. SEFrame solves the problem and achieves concurrency both between nodes and within a single node. Specifically, the transactions are protected by the SGX in the execution phase, while the trusted results are replicated in the network. To enable inter-node concurrency, a batch of transactions is divided into several micro-batches and assigned to different nodes for execution. For intra-node concurrency, a pre-execution mechanism that fetches needed data in batches is proposed to minimize the burden on the SGX. After the pre-execution, the transactions of a micro-batch are executed in the SGX with a batching optimistic concurrency control (batching OCC) protocol.

### Data Model

Existing blockchain platforms are far from convenient compared to traditional databases, as they lack the capability of modeling complex tasks in the real world. The cumbersome interfaces also prevent them from further use in business. To solve such a problem, many works aim to enable blockchains with complex semantics and easy-to-use APIs.

Since the relational model is widely used in business, many researchers and engineers try to implement relational semantics on *database-oriented blockchains*. SEBDB [41] adds relational data semantics into the blockchain platforms and supports SQL-like language as the data management interface. The block structure of SEBDB is re-designed to meet the requirement of relational semantics. Each transaction contains several system-level and user-defined application-level attributes, making it possible to maintain and store the schema as a regular relational table. FalconDB [84] is another system that explicitly supports the SQL data model. Unlike SEBDB, one FalconDB block body only consists of an arbitrary-sized transaction. And there are two attributes that record the validity time of the record to manage the history versions of data.

As for the cumbersome interfaces, BlockchainDB [83] exposes three straightforward key-value APIs, namely put, get, and verify, to the clients, while leaving the complicated primitive operations to a specifically designed storage layer. The storage layer undertakes the hideous works of reading and writing storage in a synchronized way, checking synchronization status, and computing write sets for further verification. EtherQL [43] provides two types of interfaces, namely API and REST to meet the different requirements of developers. Thus, application developers can directly use the encapsulated interfaces without fully understanding the low-level implementations. EthernityDB [82] integrates a lightweight database system with a MongoDB-like API into Ethereum by utilizing the smart contracts that are designed to process collections and documents in MongoDB.

SQL-Middleware [42] provides a different solution. Instead of modifying the underlying blockchain systems, it is a portable middleware that abstracts the blockchain into a SQL-based data management system. To be more specific, it maps each function of smart contracts into a table. When a smart contract is called, it records the structured data which is equivalent to inserting an item into the database.

### Ledger

The ledger of a blockchain records either the account state or the operations on the data in plain text. As it has been introduced in Sect. 2.1, the ledger is distributed to all the nodes of the network. In such a scenario, the secret data of one participant are also leaked among the whole system. Thus, many works try to modify the ledger to keep the privacy of sensitive data.

The first solution is to encrypt the ledger. Adkins et al. [66] designed an end-to-end encrypted system, in which data is encoded rather than stored in plaintext directly. They proposed three types of encrypted multi-map that enable efficient query and modification (including add, update, and, especially, delete) operations. The first is a list-based encryption scheme (LSX) that makes use of an append-only data store. Each value of a label is linked by its address and only the last value is stored with the label. There is a flag indicating whether it has been deleted. Then, the structure is encrypted and stored. The other two multi-maps are arranged in two dimensions, namely the tree-based scheme (TRX) and the patch-based one (PAX). As for the TRX, the list is replaced by a binary tree in the consideration of search efficiency, while in the PAX, multiple operations are packed into a patch to further improve the efficiency.

Instead of cryptographic methods, LedgerView [76] adds access control views of traditional databases to permissioned blockchains. The permission control methods can be classified from two dimensions, namely encryption-based/hash-based, and irrevocable/revocable. For the encryption-based methods, sensitive data are encrypted and stored on-chain. For the irrevocable view, the owner first creates a unique symmetric encryption key $K_i$ for each transaction $T_i$ and encrypts them, denoting them as $enc(T_i, K_i)$. Then, it produces a new symmetric key $K_V$ for the view and stores the view as a list $enc([tid_1, K_1, \ldots , tid_n, K_n], K_V)$ on the blockchain. Next, the authenticated users receive the $K_V$ from the owner and access corresponding transactions via $K_V$. For the revocable views, the lists of transactions and corresponding keys are stored separately. To revoke the user’s access, the owner simply generates a new key for the view. However, when access is revoked under the revocable views, users may still have access to information they downloaded and stored locally, but they cannot access and download further information. The hash-based methods are similar to the encryption-based ones, the difference is that data are stored off-chain and the hashes are used to verify the integrity of retrieved data.

CAPER [77] is a novel permissioned system that supports both inter- and intra-application transactions. Instead of a linear structure, the ledger in CAPER is extended to a directed acyclic graph (DAG), in which the transactions and orders are represented as vertexes and edges, respectively. In such a scenario, each application only maintains the view from its perspective, in which the order between each transaction is determined in a linear formation, while the whole ledger can be combined by all the views virtually. As for the inter-application transactions, the hashes of data from other applications are included. In this way, public records are copied on all applications, while private records of one application can only be accessed by the application to ensure privacy.

### Discussion

We make the following observations from the aforementioned representative *database-oriented blockchains*. First, techniques from traditional databases (e.g., sharding, indexing, and concurrency) benefit current *database-oriented blockchains* a lot since they have been examined and proved efficient in the past decades. It is still important to draw lessons from mature optimizing techniques. Second, there are also several *database-oriented blockchains* aim to improve those components unique to the blockchains, such as the chain-like ledger and the Byzantine resistance consensus protocol. Given the difference between blockchains and databases, such components play key roles in the functionality of *database-oriented blockchains*. Experiments show that corresponding improvements can greatly improve the performance of the system. Last, more and more *database-oriented blockchains* adopt multiple technical routes to enhance its functionality and improve performance. We can conclude that these technical routes can improve the system in various aspects from the previous part of this section. Thus, the combination of these techniques is a wise and promising way to develop further *database-oriented blockchains*.

In a word, the *database-oriented blockchains* satisfy various needs of modern data management, and the development and improvement of it are with a wide prospect.

## Blockchain-Oriented Databases

**Fig. 4:**
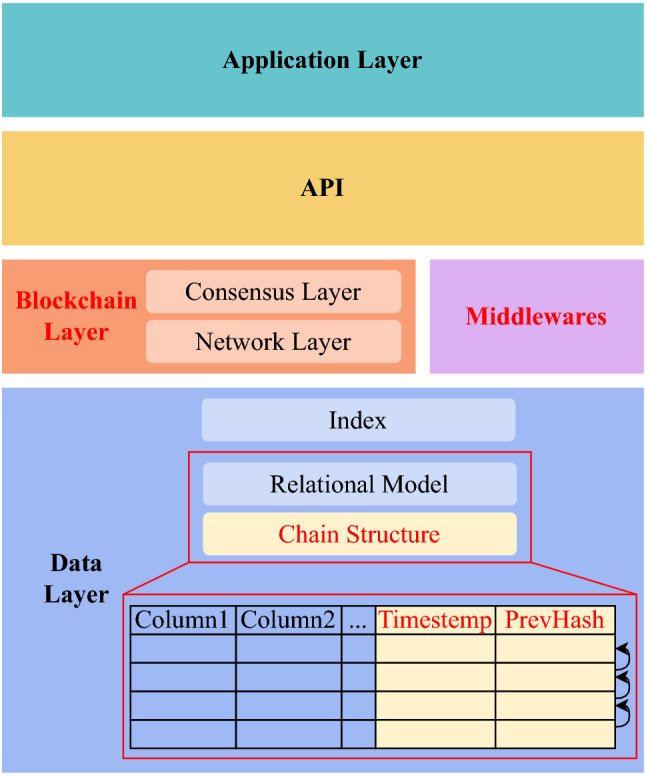
Architecture of *blockchain-oriented databases*

The *blockchain-oriented databases* take off from the database end on the blockchain-database spectrum and aim to equip the efficient and easy-to-use data management system with blockchain-powered secure guarantee. They are usually extended from mature database systems and even the already-running database instances (which are also called legacy systems). The key point of designing the *blockchain-oriented databases* is to efficiently implement the algorithms and protocols of the blockchain and minimize the impact on the base system. There are two mainstream technical routes to satisfy the requirements, namely blockchain middleware, and blockchain layer. In Fig. 4, we abstract the general architecture of the *blockchain-oriented databases* and highlight the mainly modified components.

### Blockchain Middleware

Though it is mainstream to build a blockchain from the very beginning and add database features to it in the databases community, the attempt of leveraging the existing relational databases with rich features and transactional processing capabilities to build a blockchain is also been noticed. Nathan et al. [49] studied the feasibility of such an idea and proposed blockchain relational database. They first analyzed the similarities between blockchain requirements and database features, and then implement a blockchain on PostgreSQL, an open-sourced relational database. To be more specific, several middleware and components are designed. For example, the communication middleware is used to transfer transactions and blocks, the block processor handles the received blocks and replays the transactions in the commit phase, the built-in catalog tables store related metadata, and several shared memory data structures are used to process the transactions in different isolation levels. In this way, the traditional stand-alone databases are equipped with blockchain capabilities with the cost of only 4000 lines of C code.

Since then, more and more researchers develop various blockchain middleware to enable databases with security guarantees in different aspects. Lian et al leverage the immutability of blockchain ledgers to develop a tamper-proof detection middleware for relational databases, named TRDB [48]. In TRDB, the original data are stored and processed in the relational databases, while the hash digest of each entry is replicated among the blockchain for tamper detection. There are two additional considerations, namely data privacy and supporting relational semantics in blockchains. For the first one, AES is used to encrypt the original data to protect them from the transparency nature of blockchains. To solve the second problem, TRDB concatenates both rows and columns of each table to detect illegal insertion, deletion, and modification. What’s more, TRDB caches the encrypted data with LRU strategy before it has been logged on chain to improve the data access efficiency. Thus, when querying the data, TRDB first intercepts the SQL request and executes it in the relational database, then it accesses the cache or the blockchain and subsequently compares the query results with the calculated digest. When a tamper is detected, TRDB warns of the misconduct behavior and returns the information to the user. Similar works include [110] and [111]. The former directly stores the raw data on the blockchain, while the latter concentrates on image data.

Beirami et al. [51] propose several additional built-in attributes for each relational table to simulate block headers in blockchains that support verifiable immutable transactions. The attributes include the transaction timestamp (*i*), a new table signature ($sig_i$), the previous table signature ($sig_i^{'}$), the user public key(*pubkey*), and a bit flag to indicate if the transaction is a deletion (*del*). The two signatures serve as the hash pointer of blockchain, while others are similar to the corresponding fields in block headers. Note that these attributes are calculated and inserted into the augmented tuple implicitly whenever the table is modified, which means they are transparent to the users. Given a transaction *T*, the system compares whether $enc(sig_t, pubkey) = hash(T, sig_{t-1})$ holds to verify its validity.

### Blockchain Layer

Different from the simple additions of the middleware solutions, a blockchain layer means stepping inside the underlying databases and modifying the existing components. Though it may require more effort, such a solution allows researchers to adjust the inner workflow and improve the performance of the whole system.

Blockchain PG [47] adds the blockchain function to databases to ensure data integrity, and achieve the traceability of data through trace query. It is a permissioned system that requires a CA to prove the identity of clients. The core component of the system is PostgreSQL+ (derived from PostgreSQL), whose “blockchain layer” can be further divided into four sub-layers. The user layer provides interfaces to the clients, and verifies the user’s identity with the public key; the query layer generates an optimal plan for authenticated queries to boost the query processing; the index layer provides authenticated indexes on data stored in the source layer, in which data are stored in an append-only behavior.

BigchainDB [112] is a commercial database-style distributed storage system that combines the key benefits of both distributed databases and blockchains. In fact, it is built on top of two existing RethinkDB [113] instances, namely S (stores an unordered set of transactions and serves as a backlog) and C (stores ordered list of blocks that forms a blockchain), and directly inherits the strength from databases. To enable BigchainDB with decentralized control and immutability, the authors built a blockchain layer that connects S and C with BigchainDB Consensus Algorithm (BCA) that is in charge of transaction assigning and voting.

HBasechainDB [52] adopts the same philosophy, yet it is built on Apache HBase [31], a column-based distributed database for big data. The workflow of transaction processing is quite similar to traditional blockchains such as Bitcoin – a transaction is first submitted to a transaction pool of a specific node, then the node verifies the transactions, packs all valid transactions in a block, and broadcasts to other nodes. When the majority votes for a block, it is considered to be valid and appended to the ledger, while the transactions in the invalid blocks are reassigned to other nodes randomly until it is included in the chain or removed from the system. Note that there is no need to worry about the competition between the miners and the resulting forks in practice, since HBase is of strong consistency, and the blocks are voted in the order of their timestamp. Due to the different data model, the authors designed 6 HBase tables, namely backlog, block, hbasechaindb, toVote, vote, and reference, in which backLog serves as the transaction pool and reference is an index table the maps transaction id and the content.

chainifyDB [50, 81] is a permissioned blockchain-like system built on heterogeneous database systems. The authors pointed out that blockchains and traditional DBMSs share considerable parts of their processing stack and “chainfied” existing databases by introducing a blockchain layer. To unify the heterogeneous underlying databases, a new transaction processing model called Whatever-Voting (WV) is proposed. It consists of two phases and only focuses on the results. In particular, each underlying database does whatever necessary to process the transactions and produces a digest of its behavior in the Whatever-phase, and each participant votes for the digest of W-phase in the Voting-phase. Only when an agreement is reached, the state changes are committed to a ledger by the individual organizations. chainifyDB instantiates the WV model by batching the proposed transactions and ordering them within a block. Then, the organizations use the WV model to reach a consensus on each block. For now, chainifyDB supports MySQL and PostgreSQL.

### Discussion

In this section, we review representative works about the *blockchain-oriented databases* and identify two mainstream technical routes to implement it. We can conclude from the analysis that the hash-chain feature of blockchains, along with the multi-node consensus and backup mechanism, becomes important reinforcement of traditional databases’ data integrity protection measures. The integration of blockchain features helps databases to further complete their functionality.

However, we also notice that the two technical routes have pros and cons. There exists a trade-off between flexibility and performance. In particular, building a blockchain middleware is easy and less intrusive. It can also bridge heterogeneous database instances with the same data model, which provides better portability and suits the inter-organization collaboration scenario. On the other hand, designing a blockchain layer for a specific database instance makes it possible to further optimize the components and provides higher performance. To sum up, the blockchain middleware is more friendly to the legacy systems, and the blockchain layer is more efficient. We further compare the works in Table 4.

**Table 4:** Summary of representative *blockchain-oriented databases*

Category	System	Underlying databases	Performance$^{\hbox {a}}$	Features and functionalities
Blockchain middleware	Blockchain relational Database [49]	PostgreSQL$^{\hbox {b}}$	2500 tps (Throughput)	Transform with a trivial amount of code
	TRDB [48]	MySQL $^{\hbox {b}}$	92.04% (Change in middleware execution efficiency compared to baseline)	Support tamper-proof detection
	Beirami et al. [51]	PostgreSQL$^{\hbox {b}}$	50x (Improvement of throughput)	Support verifiable immutable transaction process
Blockchain layer	Blockchain PG [47]	PostgreSQL$^{\hbox {b}}$	N/A	Transform from legacy systems
	BigchainDB [112]	RethinkDB	1,000,000 tps (Throughput)	Commercial system with high performance
	HBasechainDB [52]	HBase	5893 tps (Throughput)	Suitable for big data scenarios
	ChainifyDB [50, 81]	MySQL, PostgreSQL $^{\hbox {b}}$	5000 tps (Throughput)	Support heterogeneous database systems

$^{\hbox {a}}$We present the best result that is reported in the paper

$^{\hbox {b}}$The authors claim that their systems are suitable for any relational database, here we present the database they used in the experiments

## Hybrid Systems

The *hybrid systems* locate in the center of the blockchain-database spectrum. Different from the other fusion systems which focus on either security or performance, they are equal combinations of blockchain and database, and reach a balance between the two aspects. Though it is indeed that the *hybrid systems* are less competitive than the other fusion systems in most scenarios, the balanced and comprehensive functionality enables them to cope with the basic secure data management tasks and focus on more complex requirements. In fact, many *hybrid systems* are designed to handle complicated problems in practical scenarios, such as graph data and the conflict situation between morals and laws.

**Fig. 5:**
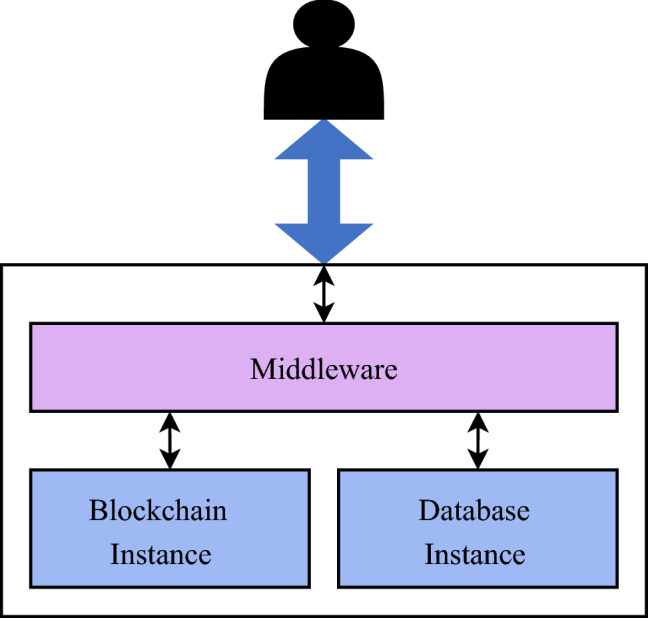
Architecture of *hybrid systems*

The integration of the *hybrid systems* usually relies on middleware to connect the existing blockchain and database instances, and we present the abstract architecture of *hybrid systems* in Fig. 5.

### Representative Systems

Instead of representing the relationship in abstract attributes, graph databases directly store and process the relationship of entities in the formation of vertexes and edges. The native graph storage and processing enable the graph databases with superior traversal performance; however, the plain KV model limits blockchains to processing such complicated data as the graph databases do. To enable the verifiable audit trail of data integrity and its modifications for information stored in a graph database, Ermolaev et al. [46] combine an Exonum [114] blockchain and Neo4j [33], the most popular graph database management system, into a single system. To be more specific, each Neo4j instance handles the data store and management, while the Exonum undertakes the role of verifiable operation log. Unlike blockchains that maintain data on the chain, the authors adopt a two-step solution that the blockchain first reaches an agreement on the modifications of graph data, and then each Neo4j instance executes the operations locally. In this way, the computation complexity and the durability of the consensus process can be greatly lowered.

In the situation of personal data management such as student data and medical records, there exists a conflict between personal privacy and public interests, i.e., stakeholders want to claim the ownership of their personal data and restrict third parties to access their data, while such a restriction may hinder the third parties to make use of these data in governance or innovation. Though blockchain and smart contract seems to be a promising solution to this problem, it is not practical since current blockchains cannot store and process such a massive amount of data. Bertram et al. [45] combine blockchain and databases to enable users to control the ownership of their private data. The system consists of three components. The core is a distributed database (e.g., BigchainDB [112]) that stores user data except identifying information. There is also a centralized MongoDB that stores user identity information. Finally, there is also a blockchain that links the two components. In particular, individual-specific smart contracts manage the map between the user identifier and his personal data. When a user decides to revoke access, he simply updates his identity in the centralized database but leaves the smart contract unchanged. Then, all third parties will lose track of this data entry while making use of the de-identified data.

The *hybrid systems* are also used to simultaneously manage data in blockchain and database platforms. This is from the observation that each platform has its solid advantage at the current stage. Thus, the most practical way is to build a combination system such that inherits both the resistance to data modification from blockchain and the query speed from the distributed databases. ChainSQL [115] is an implementation of the above scheme that ensures data integrity and fast query processing. It provides APIs that support operations in SQL and JSON format. In ChainSQL, the blockchain reaches the consensus of each transaction and stores the operations. After that, the transactions are forwarded to and executed in the database, and the actual data are also stored in the database. What’s more, ChainSQL can also serve as a disaster recovery backup since the operations are logged on a trustworthy platform, i.e., the blockchain.

The authors of MOON [44] hold another view of simultaneous data management. They believe that neither of the platforms suits all types of data, thus they aim to partition the data to either blockchain or database, and expose unified interfaces to developers and final users. In general, MOON intercepts users’ requests and redirects them to the platform that holds the data. When it comes to the situation that needs to process data from both platforms, MOON will retrieve data from the blockchain to a temporary table of the database and execute corresponding operations there to make full use of the mature data processing capability of databases. The authors also conduct a case study on clinical laboratory tests to find which platform is more suitable to hold different types of data, i.e., how to partition the data entries. They suggest that the data needed to change frequently should be stored in the database, while those persistent are more suitable to the blockchain.

### Discussion

Table 5 summarizes the representative *hybrid systems*. Although there have been numerous studies and applications of other fusion systems, we can draw from the above analysis the unique value of *hybrid systems*. By directly integrating blockchain and database instances, the *hybrid systems* acquire a balanced and sufficient ability of integrity and fast data processing from both systems, which can satisfy the needs of most application scenarios. With such a solid foundation, we can further explore some complicated problems in secure data processing (e.g., data ownership management), which may be the most important application scenario of *hybrid systems*.

We also observe that the direct integration of several instances results in a bloated and redundant system that requires more resources than the *database-oriented blockchains* or *blockchain-oriented databases*. For example, the manipulation logs on the actual data are stored both as on-chain data on the blockchain instance and as WAL logs in the database instance. How to minimize the redundancy and make full use of the instances is a promising direction for *hybrid systems* in the future.

**Table 5:** Summary of representative *hybrid systems*

System	Blockchain	Database	Research field
Ermolaev et al. [46]	Exonum	Neo4j	Graph Data
Bertram et al. [45]	Etherum	BigchainDB$^{\hbox {a}}$ (User Data), MongoDB (User Identity)	Data Ownership Management
ChainSQL [115]	Ripple	*Any*	Data Integration of Blockchain and Database
MOON [44]	BigchainDB$^{\hbox {a}}$	PostgreSQL	Data Partition between Blockchain and Database

$^{\hbox {a}}$The inconsistency comes from the underlying nature of fusion systems, depending on the authors’ focus on its functionality

## Discussion

### Comparison

Based on the above analysis, we compare the three fusion systems within different dimensions in Table 6. We can draw the conclusion that each of the fusion systems has its unique advantages and suitable scenarios. In particular, *blockchain-oriented databases* suit the scenarios that value security most and want to improve the data processing capability, while *blockchain-oriented databases* are the best choice to satisfy the security needs of the efficiency-first applications. *Hybrid systems* provide a balance between data management capabilities and blockchain benefits, making them a viable option for many use cases. Thus, the three systems are of equal importance, since the three systems satisfy different urgent demands in the data management field.

**Table 6:** A high-level comparison between three fusion systems

	Database-oriented Blockchains	Hybrid Systems	Blockchain-oriented Databases
Applicable Scenarios	Guarantee data security while Improving data processing capability	Acquire balanced properties from both sides and reduce modifications to legacy systems	Retain the data processing capability while enhancing security
Representative Systems	SEBDB [41], ForkBase [73], SlimChain [61], SE-Chain [72], BrokerChain [56]	Ermolev et al. [46], MOON [44]	TRDB [48], BigchainDB [112]
Specific Techniques	Index, Sharding, Concurrency, Data Model, Ledger	Customizable Middleware	Cryptography
Advantages	Decentralized, Data Security, Tamper-proof, Auditable	Low Coupling, Balanced Security-Performance Guarantee	High Performance, Easy-to-use, Privacy-friendly, Resource Efficient

We also observe the increasing trend in the research of *database-oriented blockchain*, which shows that people pay more attention to data security. Therefore, we suggest further studies of this aspect and expect a more competitive system based on the massive views in this field.

### Challenges and Future Works

Numerous cases have shown that there are strengths and weaknesses of blockchains and databases in the data management field, thus the integration of both systems to better undertake the task has become a promising solution in the database community. However, it is not an easy way. In this section, we present our observation on the research challenges and future opportunities in the integration of blockchains and databases, i.e., the fusion systems, from the aspects of performance, privacy, data description ability, new hardware, learning-based optimization, and application.

#### Performance

Performance is the most important feature of a data management system that is perceived by the users. Consequently, it is one of the most critical indicators to evaluate such a system. However, we have observed that there is still a huge performance gap between the fusion systems and mature commercial databases. This is due to the linear nature, one of the fundamental features of blockchain, hindering the transaction processing rate of blockchains and the succeeding fusion systems. To solve the problem, there are two parallel-but-associated targets, which affect two main operations (query and modification) in a data management system, respectively.

One is to build efficient indexes on the target data to accelerate data access. It is relatively easy for the off-chain part since the indexes of databases can achieve a satisfying performance. However, for the on-chain data, it is proved in practice that the design of new block data storage structure and corresponding indexes can effectively improve the query function and query performance. The index of on-chain data can either serve for real-time transaction verification [41, 67, 72] or improve the access efficiency of transaction history information [73–75]. In a word, a proper index of on-chain data provides not only higher real-time access efficiency in terms of performance, but also supports the traceability query based on historical data in terms of functionality.

The other is to improve the consensus mechanism, which is the key to ensuring the consistency of transaction execution among the participants, thus it has a great impact on the overall performance and application of blockchains and fusion systems. It is important to reach a balance between efficiency and consistency, but there are two main drawbacks and opportunities of current blockchain consensus mechanisms. First, the serial execution of transactions does not fully make use of the concurrency ability of modern multiprocessors, so it is a good idea to improve the concurrency of transactions [60, 63, 64]. What’s more, the abort rate in a high contention environment also encumbers the ability to process transactions. Therefore, how to effectively eliminate the conflicts remains to be researched [21, 22].

#### Privacy

In the blockchain environment, all data and transactions need to be replicated to all nodes to obtain consensus. As a consequence, sensitive data may be accessed by unauthorized third parties, and cannot be managed in the blockchain environment. The fusion systems also face such a problem. The key lies in the access control of private data. However, directly applying access control methods for databases to the blockchain will result in the hash value of each block cannot correspond to the data obtained, so users cannot verify whether the data in the chain has been tampered with. This is a difficulty that remains to be solved.

Fortunately, with the help of database techniques, several promising solutions can be further studied. For example, the system can store sensitive data in the databases (off-chain) which have better support for data privacy [41], or learn lessons from database view to manage on-chain data with access control [76]. Other technique routes include cross-chain and cryptographic protection.

#### Data Description Ability

The development of the Internet applications has spawned a variety of data forms, such as graph data and document data. Many existing studies in blockchain and database fusion systems support and extend key-value model [39, 40] and relational model [41, 42, 44, 47–51]. However, supports for the new forms of data are not common in fusion systems, and the data description capability in blockchain fusion systems needs to be improved.

For example, graph databases are a rapidly evolving field with many active research directions. Graph mining and analysis involves extracting useful information and insights from large-scale graph data. By integrating graph databases with blockchains, transactional relationships can be analyzed in a secure and decentralized manner [116].

#### New Hardware

Recently, there is a notable development of various types of hardware related to blockchains. For example, the success of Bitcoin has led to the emergence of dedicated hardware such as Field Programmable Gate Array (FPGA) and GPU, which has greatly increased the efficiency of hash computing. In turn, how to make full use of these emerging hardware in the fusion systems to better manage data is an interesting topic for the database community. Here we present several observations.

A trusted execution environment (TEE) provides an isolated memory that resists outside corruption and ensures secure computing at the hardware level, which lowers the security assumption to a certain extent. Thus, there is an opportunity to improve other aspects of blockchains, especially in the terms of performance [62, 64, 69, 70]. GPU supports parallel computing of large amounts of data, and it is a promising idea to utilize its parallel processing capability in boosting the data processing of fusion systems [117].

#### Learning-Based Optimization

Machine learning has been extensively studied over the past decades. It simulates human learning behaviors with high computing power to acquire new knowledge or skills, and has been widely applicated in database optimizations such as cost estimation, join order selection, and end-to-end optimizer [118]. We believe that it will also gain huge success in the optimization of blockchain-database fusion systems.

For example, the data distribution in sharding blockchains can greatly affect the efficiency of data access. However, current sharding systems usually adopt a naive rule such as prefix/suffix-based, which may not suit the real data distribution. In this way, machine learning-based rules can capture the pattern and boost data access [119]. Other applications include misbehavior detection of nodes [120, 121] and vulnerability analysis of smart contracts [122, 123].

#### Domain-Specific Application

The collectively maintained and tamper-resistant public ledger of blockchain systems ensures the security and reliability of the data stored in a distributed network. In addition to general-purposed data management, blockchain-database fusion systems can also bring new solutions to many specific domains. We notice that more and more people combine their original business systems with blockchains to form a domain-specific fusion system in various fields. There is a trend that leverages blockchain characteristics to solve the drawbacks of the business system, and improve the shortcomings and limitations of the blockchain system itself. However, applications in various fields have also posed more challenges.

Take finance as an example. The processing capacity of the blockchains is not enough to replace the existing centralized trading system. Therefore, it is important to improve the consensus mechanism to adapt to high throughput financial transaction applications. As for the supply chain, it is necessary to equip the system with a traceability model that fits the industrial supply chain scenario to promote verifiable data sharing in supply chain management. Other applications such as intellectual property management, asset delivery, and medical data management, also have different requirements for the fusion system.

## Conclusion

In this survey, we present the integrating trend of blockchains and traditional databases, and propose a blockchain-database spectrum to analyze the work related to the fusion systems in the field of data management. First, we classify the fusion systems into *database-oriented blockchains*, *blockchain-oriented databases*, and *hybrid systems*, and present a high-level comparison according to the different directions of their integration. Then, we review the representative fusion systems of *database-oriented blockchains*, *blockchain-oriented databases*, and *hybrid systems*. To be more specific, we review representative systems of *database-oriented blockchains* from index, protocol, data model, and ledger; we analyze blockchain middleware and blockchain layer scheme of *blockchain-oriented databases*; we also demonstrate the combination approaches and oriented research fields of different *hybrid systems*. Finally, we present a high-level comparison between the three fusion systems and our observations on the challenges and future work.

We believe that this survey demonstrates the current status and limitations of existing blockchain-related data management research and provides insight for researchers to conduct in-depth research in this area.

## Data Availability

Not applicable.
